# Progesterone Receptor Expression in the Human Enteric Nervous System

**DOI:** 10.3390/ijms27020863

**Published:** 2026-01-15

**Authors:** Naemi Kallabis, Paula Maria Neufeld, Alexandra Yurchenko, Veronika Matschke, Ralf Nettersheim, Matthias Vorgerd, Carsten Theiss, Sarah Stahlke

**Affiliations:** 1 Institute of Anatomy, Department of Cytology, Ruhr-University Bochum, 44801 Bochum, Germanyveronika.matschke@rub.de (V.M.); carsten.theiss@rub.de (C.T.); 2 International Graduate School of Neuroscience (IGSN), Ruhr-University Bochum, 44801 Bochum, Germany; 3 Visceral Surgery, BG University Hospital Bergmannsheil Bochum, 44789 Bochum, Germany; 4 Neurology, BG University Hospital Bergmannsheil Bochum, 44789 Bochum, Germany

**Keywords:** enteric nervous system (ENS), progesterone receptors, gut–brain axis, human intestine, neurodegenerative disease, steroid hormones, sex differences, age differences

## Abstract

The enteric nervous system (ENS) is a critical component of the gut–brain axis, playing a pivotal role in gastrointestinal homeostasis and systemic health. Emerging evidence suggests that ENS dysfunction precedes central neurodegenerative disorders. Progesterone, known for its neuroprotective and anti-inflammatory properties in the central nervous system (CNS), has received growing attention for its potential role in ENS physiology. This study aimed to map the expression of nuclear and membrane-bound progesterone receptors in the human ENS, considering regional intestinal, sex, and age variations. Immunofluorescence and Reverse Transcription-Polymerase Chain Reaction (RT-PCR) were used to evaluate receptor distribution in anatomically distinct intestinal regions. Consistent expression of classical nuclear progesterone receptors (PR-A/B) and the non-classical Progesterone receptor membrane component 1 (PGRMC1) in myenteric ganglion cells across all intestinal segments was observed. RT-PCR confirmed the expression of PR-A/B, PGRMC1, mPRα, and mPRβ, with regional variations. Sex-specific patterns were evident along with age-related downregulation. Our findings provide a detailed characterization of progesterone receptor expression in human ENS, highlighting sex- and age-dependent regulation. The identification of progesterone signaling within the myenteric plexus suggests a hormonal influence in gut–brain communication. Targeting ENS progesterone receptors may open novel therapeutic avenues to modulate neurodegenerative CNS disorders via peripheral intervention along the gut–brain axis.

## 1. Introduction

The enteric nervous system (ENS), often referred to as the “second brain,” is a complex, autonomous network of neurons and glial cells embedded in the gastrointestinal tract [1]. While it coordinates digestive function locally, it is also a key component of the gut–brain axis (GBA), a bidirectional communication system linking the ENS with the central nervous system (CNS) via neural, immune, endocrine, and microbial pathways [2]. This axis is crucial for maintaining homeostasis and is increasingly implicated in the pathogenesis of neurodevelopmental, neurodegenerative, and psychiatric disorders [3].

The gut microbiota and immune signals play a pivotal role in gut–brain communication, influencing both gastrointestinal functions and CNS health. The gut microbiota, comprising trillions of microorganisms, produces a myriad of neuroactive molecules that can modulate brain function and behavior [4]. Additionally, immune signals, such as cytokines and chemokines, can cross the blood–brain barrier and affect CNS function, highlighting the intricate interplay between the gut and the brain [5].

Notably, the ENS is often one of the first sites affected in neurodegenerative diseases, such as Parkinson’s disease (PD) [6]. PD, a widespread movement disorder characterized by symptoms such as bradykinesia, rigidity, resting tremor, and postural instability, results from a dopamine deficiency, with various causes. After Alzheimer’s disease, it is the second most common neurodegenerative disorder, affecting millions worldwide [7]. Recent epidemiological studies indicate it is the fastest-growing neurological condition in terms of both prevalence and mortality, highlighting its increasing health burden [8].

The Braak hypothesis suggests that PD often originates in the peripheral nervous system (PNS), particularly in the gut or olfactory system, and spreads to the CNS. This is supported by studies of the prodromal phase, where patients experience non-motor symptoms such as constipation, depression, anxiety, orthostatic hypotension, and erectile dysfunction before the onset of motor symptoms. These early signs suggest that the pathology of PD may affect peripheral and autonomic systems long before motor deficits become clinically evident [9,10,11]. In accordance with the Braak hypothesis, α-synuclein pathology may originate in the gastrointestinal system and disseminate to the brain via transsynaptic propagation along the vagus nerve [12]. This underscores the significance of peripheral neural systems in central neurodegeneration.

Recent studies have identified a gender-specific susceptibility to PD, with men being more frequently and severely affected than women [13]. These differences tend to diminish with age, suggesting that hormonal shifts, such as those occurring during menopause, might modulate disease vulnerability [14]. The start of menopause in women typically occurs between ages 45 and 55, with the average age in most countries around 51 to 52 years old [15]. The menopausal transition, known as perimenopause, often begins in the late 40s and can last for several years before menopause is reached [16]. This makes it necessary to explore steroid hormone signaling not only by sex, but across the lifespan.

Steroid hormones, particularly progesterone, have attracted attention for their neuroprotective, anti-inflammatory, and regenerative functions within both the CNS and ENS [17,18]. Progesterone exerts its effects via nuclear receptors (PR-A, PR-B) and membrane-associated receptors (mPRα, mPRβ, PGRMC1), mediating both genomic and non-genomic signaling [19]. While the expression and function of these receptors have been extensively studied in central structures, their distribution in the human ENS, particularly in anatomically resolved contexts, remains poorly understood.

Another important factor is obesity (BMI ≥ 30), which leads to significant disturbances in sex hormone regulation in both women and men, with women experiencing disrupted ovulation and lower progesterone due to increased peripheral estrogen production from adipose tissue, while men typically exhibit decreased testosterone and altered estrogen-to-androgen ratios [20].

Despite the acknowledged relevance of hormonal modulation, a detailed characterization of progesterone receptor expression in the ENS across different gut regions and demographic backgrounds is lacking. The anatomical organization of the ENS, including regional differences in extrinsic innervation, might be critical for understanding site-specific vulnerability and responsiveness to hormonal signals. In this context, the Cannon–Böhm point is a relevant anatomical landmark as it marks the transition zone in the midgut where parasympathetic innervation shifts from the vagus nerve to the pelvic splanchnic nerves, resulting in a distinct change in neurochemical environment and enteric circuitry [21]. Understanding the expression patterns of progesterone receptors in the ENS could provide insights into novel therapeutic strategies for neurodegenerative diseases.

In this study, we analyzed the expression patterns of nuclear and membrane-bound progesterone receptors in the human ENS, considering regional variation, sex, age, BMI, and diet. Our findings aim to provide a foundation for future functional investigations into neuroendocrine modulation of the gut and its relevance for neurodegeneration and individualized medicine.

## 2. Results

Our study aimed to analyze the expression patterns of nuclear and membrane-bound progesterone receptors in the human enteric nervous system (ENS), considering regional variation, sex, age, BMI, and diet. The results provide a comprehensive overview of the distribution and potential functional implications of these receptors in the ENS.

### 2.1. PGRMC1 and PR-A/B Are Expressed in the Human Gut

**Figure 1:**
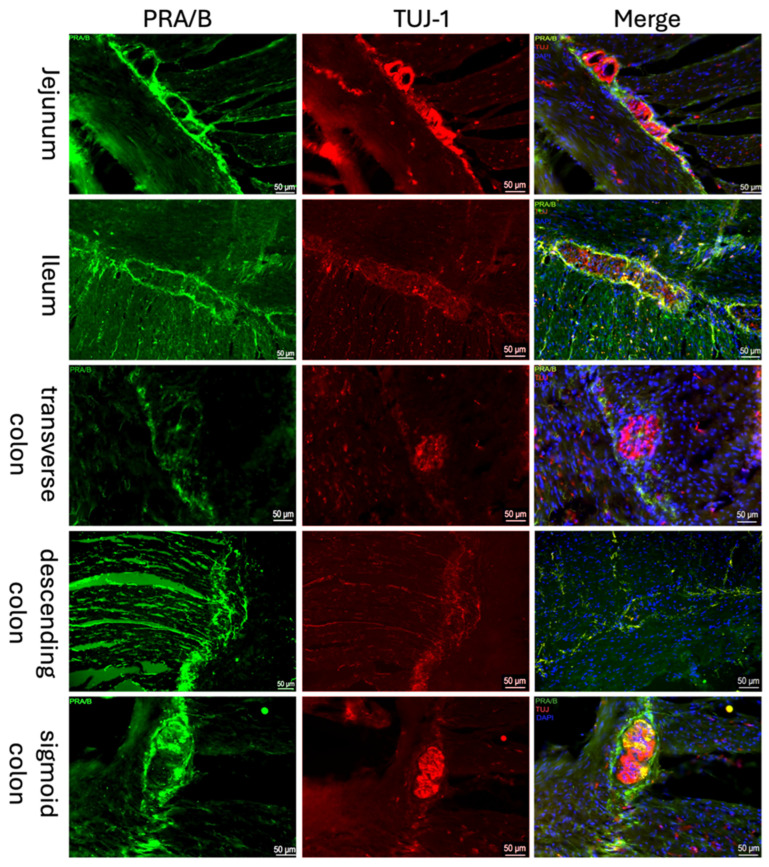
Immunofluorescence staining of myenteric ganglia. Immunofluorescence staining of the myenteric plexus using antibodies against PRA/B (first column, green) was conducted in various intestinal regions. To visualize the myenteric plexus, co-staining against TUJ-1 (second column, red) was performed. The third column shows a merged image of PRA/B (green), TUJ-1 (red) and DAPI (blue, cell nuclei). The samples were obtained from the jejunum of a male patient, the ileum of a female patient, the transverse colon of a male patient, the descending colon of a female patient, and the sigmoid colon of another female patient.

**Figure 2:**
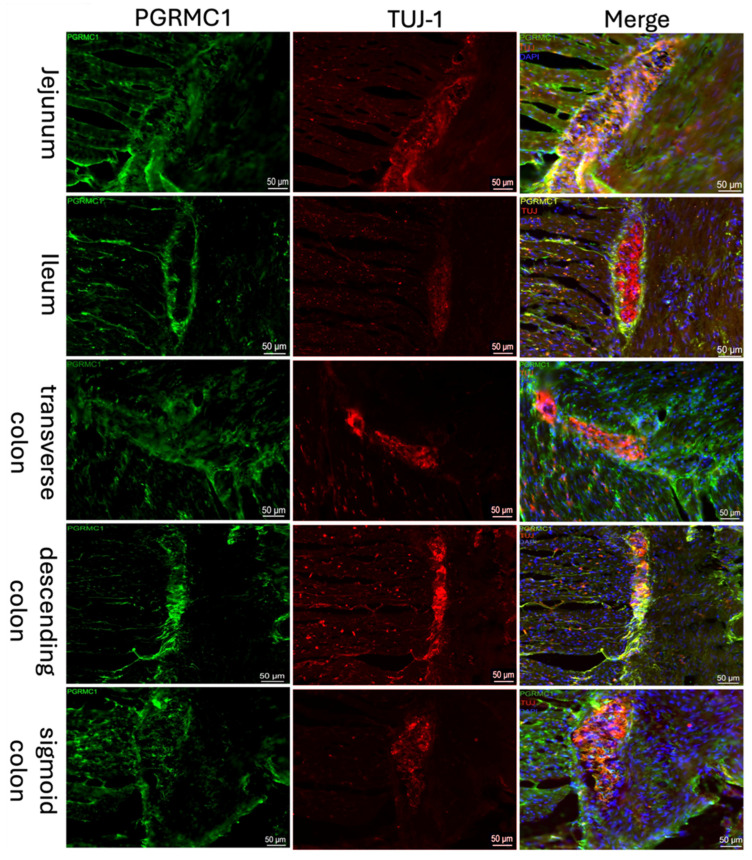
Immunofluorescence staining of the myenteric plexus using antibodies against PGRMC1 (first column, green) in various sections of the intestine. To visualize the myenteric plexus, co-staining against TUJ-1 (second column, red) was performed. The third column shows a merged image of PGRMC1 (green), TUJ-1 (red) and DAPI (blue, cell nuclei). Samples were taken from the jejunum of a male patient, the ileum of a female patient, the transverse colon of a male patient, the descending colon of a female patient, and the sigmoid colon of another female patient.

Both the classical nuclear progesterone receptor PR-A/B (Figure 1) and the non-classical progesterone receptor “Progesterone Receptor Membrane Component 1” (PGRMC1; Figure 2) were detected immunohistochemically in all sections of the intestine. A well-defined fluorescence signal was observed in the ganglion cells of the myenteric plexus (Tuj-1 positive; red Figure 2, Figure 1) of the intestine. The expression could be found throughout the myenteric plexus and at its borders, but was not exclusively in this area. It was also expressed between and parallel to muscle fibers. Samples from the jejunum, ileum, and colon showed similar distribution and intensity of PGRMC1 and PR-A/B, with consistent signal localization in ENS ganglion cells across all subjects.

**Figure 3:**
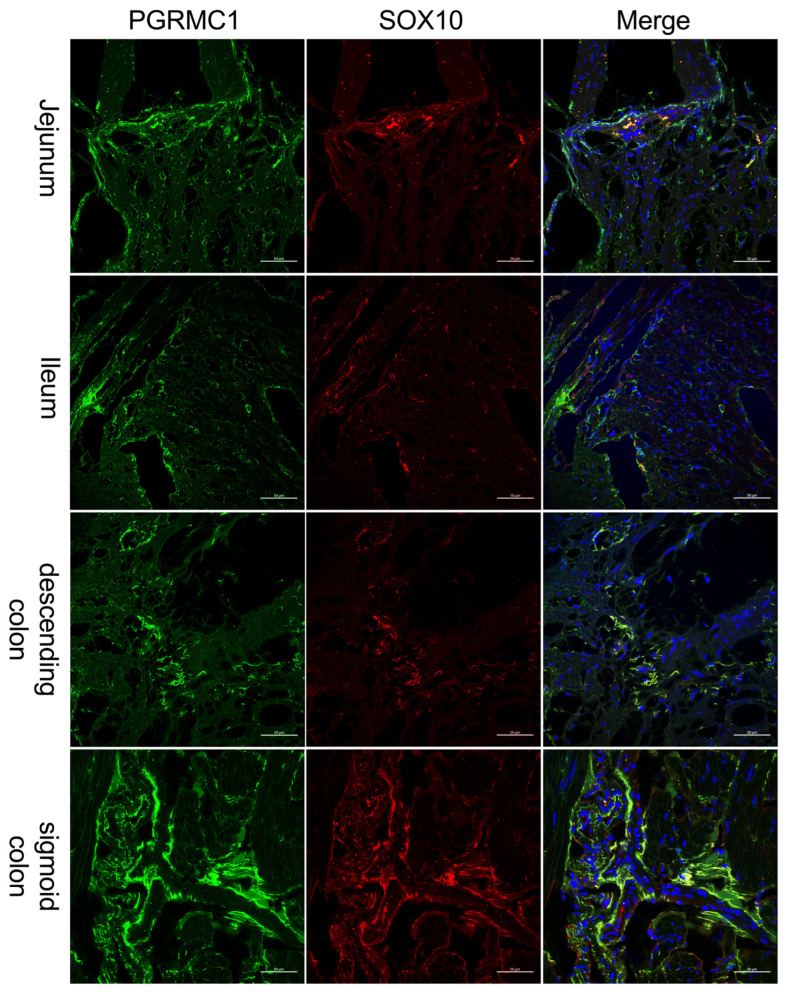
Immunofluorescence staining of the myenteric plexus using antibodies against PGRMC1 (first column, green) in various sections of the intestine. To visualize co-localization, co-staining against SOX10 (second column, red) was performed. The third column shows a merged image of PGRMC1 (green), SOX10 (red) and DAPI (blue, cell nuclei). Samples were taken from the jejunum of a female patient, the ileum of a female patient, the descending colon of a female patient, and the sigmoid colon of a male patient. The scale bar indicates 50 µm for reference.

**Figure 4:**
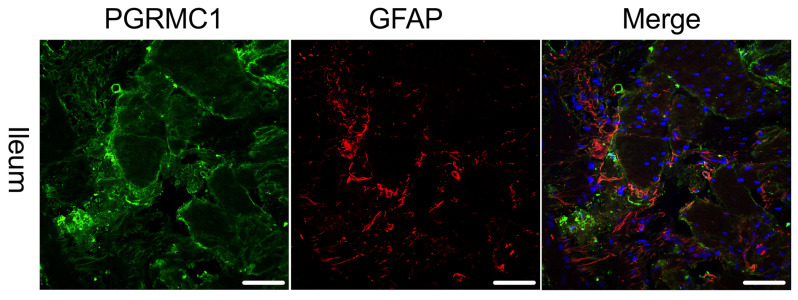
Immunofluorescence staining of the myenteric plexus using antibodies against PGRMC1 (first column, green) in the ileum of a female patient. To visualize co-localization, co-staining against GFAP (second column, red) was performed. The third column shows a merged image of PGRMC1 (green), GFAP (red) and DAPI (blue, cell nuclei). The merge shows no co-localization of PGRMC1 with GFAP. The scale bar indicates 50 µm for reference.

As receptor expression was not restricted to Tuj-1-positive cells, co-expression of PGRMC1 with SOX10 (Figure 3)-positive cells, as a marker for ENS progenitors and enteric glia, as well as GFAP (Figure 4) as a glial cell marker, was also investigated. Figure 3 shows that for SOX10, a partial co-expression with PGRMC1 was observed, whereas no overlap was found for GFAP; therefore, only the ileum is shown as an example in Figure 4.

### 2.2. RT-PCR Results

RT-PCR analysis confirmed, in addition to the immunohistochemical stainings, the expression of PR-A/B and PGRMC1, but also the membrane-bound progesterone receptors, membrane progestin receptors alpha and beta (mPRα, mPRβ), which are normalized to the housekeepers (β-Actin and GAPDH).

#### 2.2.1. Correlation Between Progesterone Receptors and Enteric Nervous System Cells

**Figure 5:**
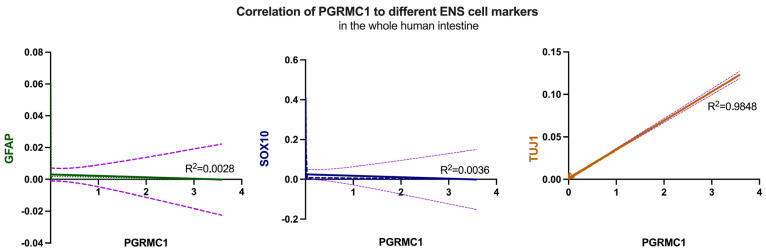
Correlation of GFAP (left, green), Sox10 (middle, blue) and Tuj-1 (right, orange) with PGRMC1, respectively, over the whole human intestine. Despite Tuj-1, no correlation could be detected. The corresponding R^2^ values are indicated in the graphs, with 0.0028 for GFAP, 0.0036 for Sox10 and 0.9848 for Tuj-1. The lilac dotted line in all graphs shows the deviation.

To further characterize the observed expression patterns within the enteric nervous system, the expression of GFAP (glial fibrillary acidic protein), an important structural protein of astrocytes, SOX10 as an ENS precursor marker, and Tuj-1 as a neuronal marker were investigated. Initially, the expression levels of the selected markers were correlated with those of the receptors under investigation in the different sections of the intestine. Linear regression analyses were performed, and the resulting graphs and R^2^ values were visualized. Figure 5 shows the plotted results of these analyses; no correlation could be detected for GFAP (R^2^ = 0.0028) in line with the previous results obtained by immunohistochemistry, also Sox10 (middle) showed no correlation (R^2^ = 0.0036) considering the entire analyzed intestine. In contrast to these findings but mirroring immunohistochemistry, there is a strong positive correlation (R^2^ = 0.9848) for Tuj-1 throughout the entire intestine.

**Figure 6:**
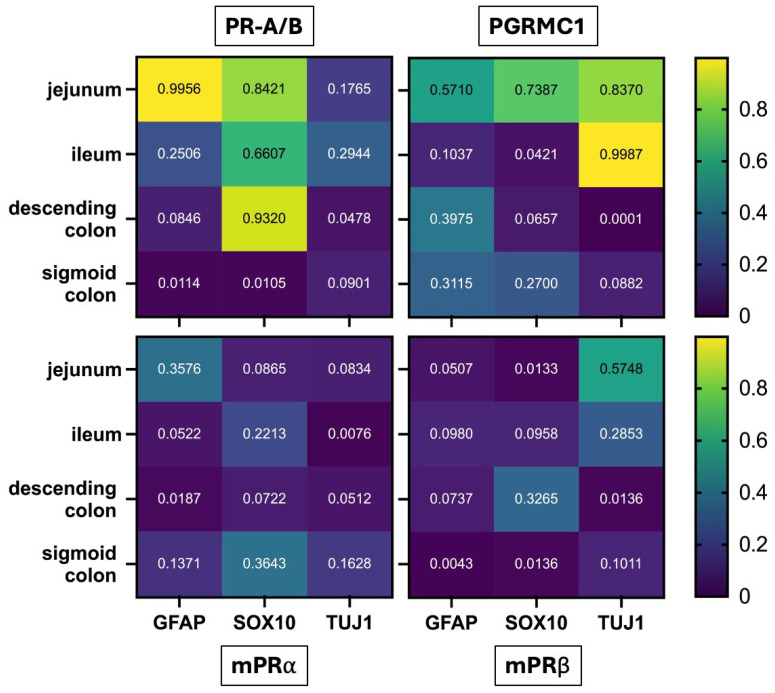
Heatmap showing R^2^ values from linear regression analyses correlating progesterone receptor expression (PR-A/B, PGRMC1, mPRα, mPRβ) with neural and glial markers (GFAP, SOX10, TUJ1) across different intestinal regions (jejunum, ileum, descending colon, sigmoid colon). Color intensity reflects R^2^ values from 0 (dark blue) to 1 (yellow), with values > 0.8 indicating relevant correlations. Notable correlations include PR-A/B with GFAP in the jejunum (R^2^ = 0.9956) and SOX10 in both the jejunum (R^2^ = 0.8421) and descending colon (R^2^ = 0.9320), as well as PGRMC1 with TUJ1 in the jejunum (R^2^ = 0.8370) and ileum (R^2^ = 0.9987).

In a second approach, a deeper look into the different areas was taken; therefore, the obtained R^2^ values were plotted in a heatmap covering the four intestinal regions (jejunum, ileum, descending colon and sigmoid colon). The color scale ranged from dark blue (R^2^ = 0) to yellow (R^2^ = 1), with values above 0.8 indicating meaningful correlations (Figure 6).

The strongest associations were observed for PR-A/B, which showed a strong correlation with GFAP in the jejunum (R^2^ = 0.9956), suggesting a pronounced link between PR-A/B expression and enteric glial cells in this region. A substantial correlation was also found between PR-A/B and SOX10 in the jejunum (R^2^ = 0.8421) and, notably, in the descending colon (R^2^ = 0.9320), indicating region-specific relationships with glial or neural crest–derived cell populations.

For PGRMC1, positive correlations with the neuronal marker TUJ-1 emerged in two regions: the jejunum (R^2^ = 0.8370) and the ileum (R^2^ = 0.9987). No relevant correlations were detected for mPRα or mPRβ with any of the assessed markers in any intestinal region.

Together, these results highlight receptor- and region-specific patterns suggesting that classical and non-classical progesterone receptors may differentially associate with neuronal and glial subsets of the enteric nervous system.

#### 2.2.2. Regional Variation in Progesterone Receptor Expression

**Figure 7:**
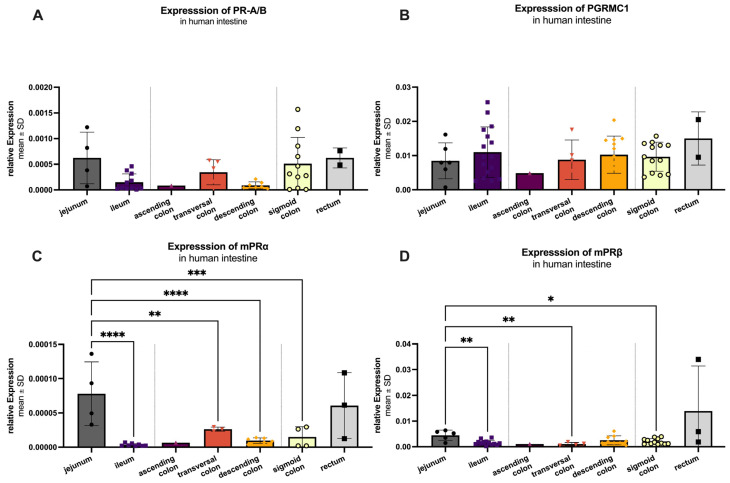
RT-qPCR analysis of PR-A/B (**A**), PGRMC1 (**B**), mPRα (**C**) and mPRβ (**D**) in the human intestine. mRNA could be detected in all analyzed areas: jejunum (*n* = 4–5), ileum (*n* = 13), ascending colon (*n* = 1), transversal colon (*n* = 3–4), descending colon (*n* = 6–9), sigmoid colon (*n* = 11–13) and rectum (*n* = 2–3). Ascending colon and rectum were excluded from statistical analyses due to small *n* values. Of the remaining groups, expression is significantly highest in the jejunum for all markers except PGRMC1, which is highest in ileum but not significantly different from the other regions. Significant differences are indicated by an asterisk (*), with significant levels of: * *p* < 0.05, ** *p* < 0.01, *** *p* < 0.001 and **** *p* < 0.0001.

The regional expression pattern of the receptors was analyzed from jejunum (dark gray), ileum (purple), colon (ascendens (dark pink), transversum (light pink) and descendens (orange), sigmoid (yellow)) and rectum (light gray; Figure 7). Results are plotted as mean 2^−Δct^ with standard deviation (SD).

Among all groups, PGRMC1 (Figure 7B) exhibited the highest mRNA expression with a mean value of 0.01008 ± 0.00576, followed by mPRβ (0.00232 ± 0.00170; Figure 7D). The classic receptor (PR-A/B; Figure 7A) was expressed at 0.00041 ± 0.00048, while mPRα (Figure 7C) showed the lowest expression levels (0.00003 ± 0.00004). Summarized PGRMC1 expression was significantly higher (*p* < 0.001) compared to the other three receptors, while mPRβ was significantly higher expressed as PR-A/B (*p* = 0.031) and mPRα (*p* = 0.011). For PR-A/B (Figure 7A), no significant difference in expression was observed between the analyzed intestine sections. The same was true for PGRMC1 (Figure 7B). Significant regional variations were found in the expression of mPRα (Figure 7C). It showed a significant difference between the highest expression in jejunum (0.00008 ± 0.00005) compared to ileum (*p* < 0.0001), transversal colon (*p* = 0.0068), descending colon (*p* < 0.0001) and sigma (*p* = 0.0005). The expression pattern of mPRβ (Figure 7D) mirrored that of mPRα, with significant differences between the jejunum (highest expression 0.00449 ± 0.00199) and the ileum (*p* = 0.0015), transversal colon (*p* = 0.0040), and sigma (*p* = 0.0114).

#### 2.2.3. Sex-Specific Differences in Progesterone Receptor Expression

**Figure 8:**
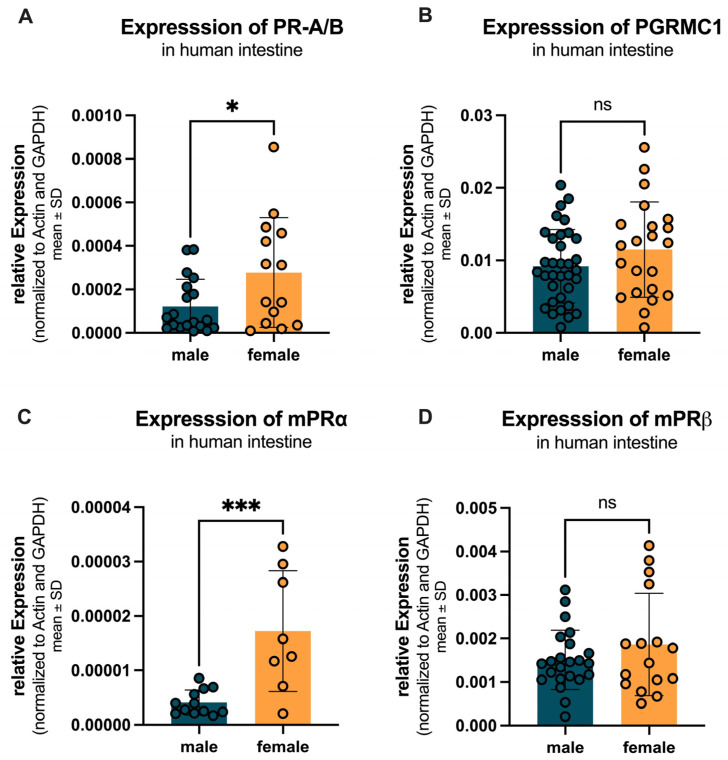
Sex-specific differences in progesterone receptor expression across the entire intestine. (**A**) PR-A/B expression is significantly higher in females (yellow) compared to males (teal). (**B**) PGRMC1 expression is not changed in females compared to males. (**C**) mPRα expression is significantly higher in females compared to males. (**D**) mPRβ expression shows no significant difference between females and males. Significant differences indicated by * *p* < 0.05, and *** *p* < 0.001; ns = not significant.

Sex-specific differences in the expression of progesterone receptors were analyzed across the entire intestine, including jejunum, ileum, colon (ascendence, transversum, descendent, sigmoid) and rectum. Figure 8A shows that PR-A/B expression was significantly higher in females (0.00028 ± 0.00025) compared to males (0.00012 ± 0.00012) with a *p*-value of 0.026. PGRMC1 expression was not significantly changed in females (0.0115 ± 0.0066) compared to males (0.0092 ± 0.0051) as depicted in Figure 8B, with a *p*-value of 0.002. Figure 8C illustrates that mPRα expression was also significantly higher in females (0.000017 ± 0.000011) compared to males (0.000004 ± 0.000002), with a *p*-value of 0.0008. In contrast, Figure 8D shows no significant difference in mPRβ expression between females (0.0019 ± 0.0012) and males (0.0015 ± 0.0007).

**Figure 9:**
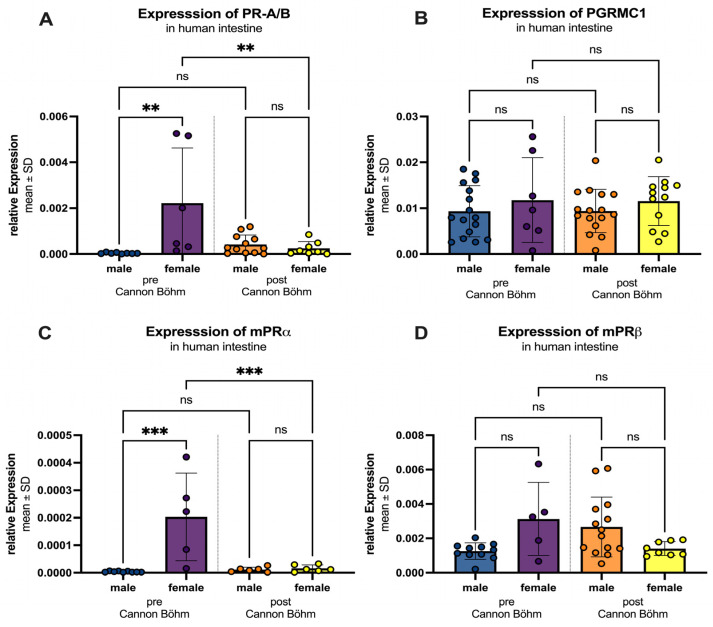
Regional and sex-specific differences in progesterone receptor expression. Three segments are differentiated: “pre Cannon–Böhm” male (blue) and female (purple) and “post Cannon–Böhm” with male (orange) and female (yellow). (**A**) PR-A/B expression is significantly higher in females compared to males before the Cannon–Böhm point, but shows no significant difference after the Cannon–Böhm point. Also expression in females is significantly higher before the Cannon–Böhm point compared to females after the Cannon–Böhm point. (**B**) PGRMC1 expression shows no significant differences in females compared to males before or after the Cannon–Böhm point. (**C**) mPRα expression is significantly higher in females compared to males before the Cannon–Böhm point, but shows no significant difference after the Cannon–Böhm point. Expression significantly decreases in females after the Cannon–Böhm point. (**D**) mPRβ expression shows a higher trend in females compared to males before the Cannon–Böhm point, although the difference is not statistically significant, and no significant difference is observed after the Cannon–Böhm point. Data are presented as mean ± SD, with significant differences indicated by ** *p* < 0.01, and *** *p* < 0.001; ns = not significant.

If the intestine is further divided into the different innervations, before the Cannon–Böhm point (jejunum, ileum and ascending colon) with vagal innervation and after the Cannon–Böhm point (descending colon, sigmoid colon and rectum), some more details came up. Figure 9 illustrates the regional and sex-specific differences in progesterone receptor expression, divided by the Cannon–Böhm point. For all four receptors (PR-A/B, PGRMC1, mPRα, and mPRβ), a higher expression was observed in females compared to males in the segment before the Cannon–Böhm point. Specifically, PR-A/B expression was significantly higher in females (0.0022 ± 0.0024) compared to males (0.0004 ± 0.0003) with a *p*-value of 0.0017. But after the Cannon–Böhm point expression in females reduced significantly (*p* = 0.0048). PGRMC1 expression was slightly elevated in females (0.0118 ± 0.0092) compared to males (0.0093 ± 0.0056), but not statistically significant. mPRα expression was significantly higher in females (0.00020 ± 0.00016) compared to males (0.000004 ± 0.000002) with a *p*-value of 0.0001, again with significant downregulation after Cannon–Böhm point (*p* = 0.0007). mPRβ expression showed a higher expression in females (0.0031 ± 0.0021) compared to males (0.0013 ± 0.0005); the difference was not statistically significant (*p* = 0.0544). In contrast, no significant differences were observed between females and males in the segments after the Cannon–Böhm point for any of the receptors (PR-A/B: females 0.00025 ± 0.00029 vs. males 0.0004 ± 0.0004, *p* = 0.993; PGRMC1: females 0.0116 ± 0.0053 vs. males 0.0094 ± 0.0047, *p* = 0.812; mPRα: females 0.000016 ± 0.000012 vs. males 0.000011 ± 0.000009, *p* > 0.999; mPRβ: females 0.0014 ± 0.0004 vs. males 0.0027 ± 0.0017, *p* = 0.154).

#### 2.2.4. Age-Related Changes in Progesterone Receptor Expression

**Figure 10:**
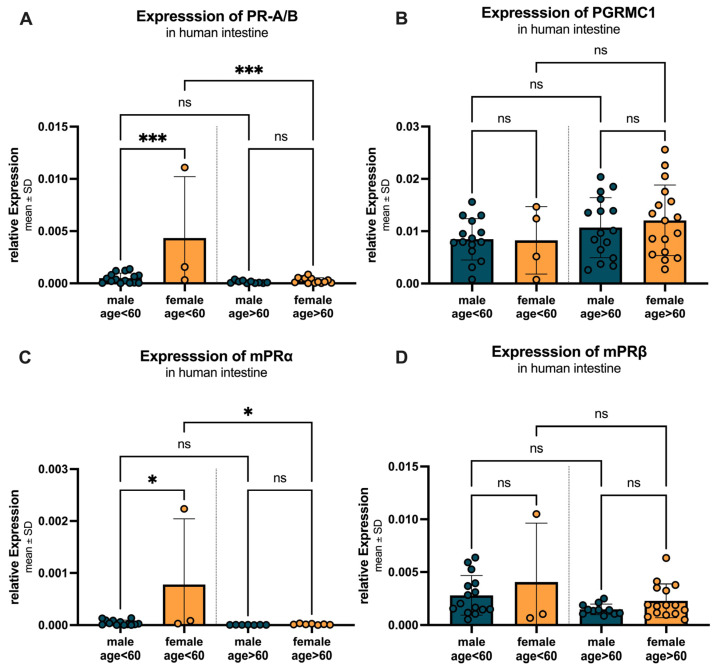
Age- and sex-specific differences in progesterone receptor expression. (**A**) PR-A/B expression is significantly higher in females under 60 years compared to males, with a significant decrease in expression in females over 60 years. (**B**) PGRMC1 expression shows no changes in expression in females compared to males, with an increase in expression observed in both sexes over 60 years. (**C**) mPRα expression is significantly higher in females under 60 years compared to males, with a significant decrease in expression in females over 60 years. (**D**) mPRβ expression is higher in females compared to males, with a decrease in expression observed in both sexes over 60 years. Data are presented as mean ± SD, with significant differences indicated by * *p* < 0.05, and *** *p* < 0.001, ns = not significant.

Figure 10 illustrates the age- and sex-specific differences in progesterone receptor expression, divided into two age groups: under 60 years and over 60 years. For PR-A/B (Figure 10A), females under 60 years showed a significantly higher expression (0.0043 ± 0.0059) compared to males (0.0005 ± 0.0005) with a *p*-value < 0.0005. In individuals over 60 years, no significant difference was observed between females (0.00027 ± 0.00026) and males (0.00013 ± 0.00012) (*p* = 0.9985). Within the female group, PR-A/B expression significantly decreased in individuals over 60 years compared to those under 60 years, with a *p*-value = 0.0003. In contrast, the decrease in expression in males over 60 years compared to those under 60 years was not significant (*p* = 0.947). A similar pattern was observed for mPRα (Figure 10C), with significantly higher expression in females under 60 years (0.00078 ± 0.00126) compared to males (0.00005 ± 0.00005; *p* = 0.0118), no significant difference in individuals over 60 years (females: 0.000015 ± 0.000011 vs. males: 0.000005 ± 0.000002, *p* = 0.999), and a significant decrease in expression in females over 60 years compared to those under 60 years (*p* = 0.0153). For PGRMC1 (Figure 10B), no statistically significant differences between females compared to males could be found in neither the individuals below (*p* > 0.999) nor above (*p* = 0.931) the age of 60. The expression tended to increase with age (male over 60: 0.0107 ± 0.0057 vs. females over 60: 0.0121 ± 0.0067). For mPRβ (Figure 10D), females showed higher expression levels compared to males, with a decrease in expression observed in both sexes over 60 years (females: under 60: 0.0041 ± 0.0056 vs. over 60: 0.0023 ± 0.0016; males: under 60: 0.0082 ± 0.0019 vs. over 60: 0.0015 ± 0.0005), both not statistically significant.

#### 2.2.5. Impact of BMI and Diet on Progesterone Receptor Expression

**Figure 11:**
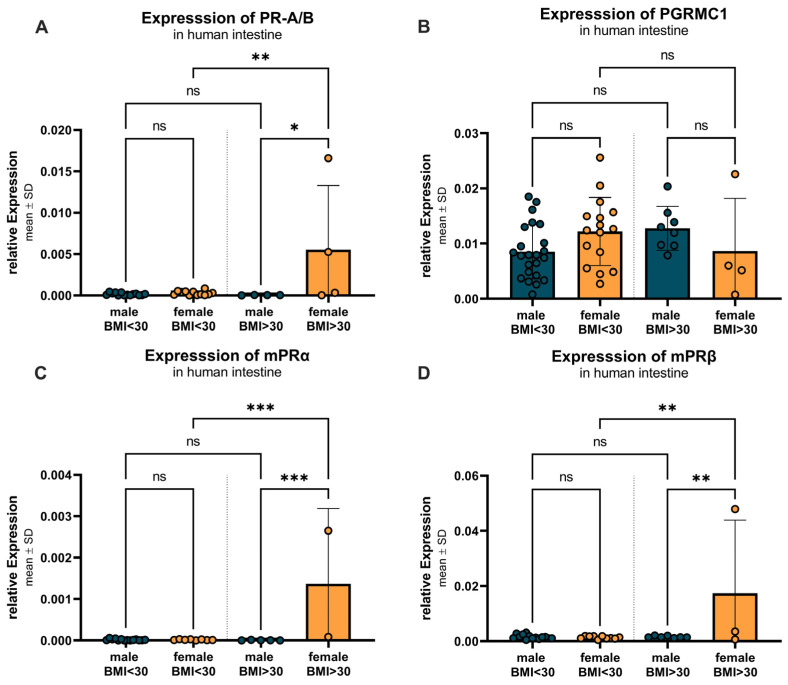
BMI- and sex-specific differences in progesterone receptor expression. (**A**) PR-A/B expression shows no significant differences between males and females with BMI under 30, but upregulation in females with BMI over 30 compared to males and females with BMI under 30. (**B**) PGRMC1 expression is not altered between groups and sexes. (**C**) mPRα expression shows no significant differences between males and females with BMI < 30, but higher expression in females with BMI > 30 compared to corresponding males and females with BMI < 30. (**D**) mPRβ expression shows no significant differences between the sexes in the BMI < 30 group, but again higher expression in females with BMI > 30 compared to corresponding males and females with BMI < 30. Data are presented as mean ± SD, with significant differences indicated by * *p* < 0.05, ** *p* < 0.01, and *** *p* < 0.001; ns = not significant.

Figure 11 examines the BMI- and sex-specific differences in progesterone receptor expression, divided into two BMI groups: BMI < 30 and BMI > 30. For PR-A/B (Figure 11A), a significant (*p* = 0.0034) increase in expression was observed between females with lower (0.0003 ± 0.0003) compared to higher BMI (0.0055 ± 0.0077), as well as a significant (*p* = 0.0139) higher expression in females compared to males (0.00004 ± 0.00002) in the higher BMI group. For PGRMC1 (Figure 11B), females with a BMI < 30 showed similar expression compared to males in both groups. The expression pattern of mPRα (Figure 11C) mirrored that of PR-A/B, with no significant differences between males and females with a BMI of less than 30. However, within the female group, mPRα expression significantly increased in individuals with a BMI > 30 (0.0014 ± 0.0018) compared to those with a BMI < 30 (0.000017 ± 0.000011), with a *p*-value of 0.0003. The females with a BMI higher than 30 also showed a significantly (*p* = 0.0006) higher expression compared to the corresponding males (0.000005 ± 0.000005). For mPRβ (Figure 11D), a similar expression pattern was observed between the groups. With a significantly higher expression in females with higher BMI (0.0174 ± 0.0265) compared to their male counterparts (*p* = 0.0092; (0.0016 ± 0.0004) as well as compared to the females with lower BMI (*p* = 0.0053; (0.0014 ± 0.0005).

Additionally, the impact of dietary patterns on progesterone receptor expression was investigated. However, due to the limited number of samples (*n* = 4) from only female vegetarians, no conclusive statements could be made at this time. Further studies with a larger sample size are needed to elucidate the potential effects of dietary patterns on progesterone receptor expression.

## 3. Discussion

Our study provides compelling evidence for the complex role of progesterone receptors in the human enteric nervous system (ENS), revealing significant gender-specific, age-related, and regional variations in their expression. These findings not only advance our understanding of ENS physiology but also open new avenues for exploring hormonal modulation in neurodegenerative diseases, particularly PD.

### 3.1. Progesterone Receptor Expression Patterns

Immunofluorescence staining revealed distinct expression patterns of progesterone receptors in the ENS, co expressed with both neuronal and glial lineage cells. The higher expression of PGRMC1 in females suggests a gender-specific regulatory mechanism that may be crucial for maintaining gut homeostasis. The regional differences in mPRα and mPRβ expression between large and small intestines point to specialized functions in different gut segments, potentially reflecting the unique neurochemical environments and physiological demands of these regions.

The partly downregulated receptor levels in females behind the Cannon–Böhm point may reflect the unique neurochemical environment in this transition zone, where parasympathetic innervation shifts from the vagus nerve to the pelvic splanchnic nerves. This anatomical landmark could be particularly relevant for understanding site-specific vulnerability and responsiveness to hormonal signals in the gut.

The age-related decline in PR-A/B and mPRα expression, particularly in female individuals over 60, aligns with known hormonal changes during aging [22]. This decline may contribute to the increased susceptibility to gastrointestinal disorders observed in older populations [23]. The stable expression of PGRMC1 across age groups suggests this receptor may respond to different regulatory mechanisms, potentially involving oxidative stress or inflammatory pathways, as demonstrated by [24].

The presence of progesterone receptors on Beta-III-Tubulin-positive neurons indicates a direct influence on neuronal function. Beta-III-Tubulin is a specific marker for neuronal cells and plays a crucial role in axonal transport and structural integrity [25,26]. The expression of progesterone receptors might exert specific effects on the neurons of the ENS, potentially modulating gut motility, sensory processing, and neuroimmune interactions [27]. As a co-expression of progesterone receptors with Sox-10-positive cells was also found, this might additionally hint at enteric glia cell contribution. This finding is particularly interesting in the context of inflammation, immunological, and degenerative processes, as enteric glia play a crucial role in maintaining gut homeostasis and responding to injury or inflammation. While our study provides evidence for the expression of progesterone receptors in the myenteric plexus, the precise cellular localization of these receptors remains to be fully elucidated. Our double staining experiments revealed a co-expression of PGRMC1 with Sox-10 as well as Tuj-1-positive cells, suggesting that PGRMC1 may be expressed in enteric glia as well as neurons. However, the lack of co-expression with GFAP indicates that not all glial cells express PGRMC1. These findings highlight the complex cellular distribution of progesterone receptors in the ENS and the need for further investigation to fully understand their functional roles.

### 3.2. Gender-Specific Adaptations

The elevated expression of PR-A/B and mPRα in the females pre Cannon–Böhm point suggests an adaptive mechanism for hormonal fluctuations, e.g., during the menstrual cycle and pregnancy. Progesterone’s role in increasing occludin expression [28] and modulating intestinal muscle contraction [29] highlights its importance in maintaining gut barrier function and motility. These findings suggest that progesterone receptors may play a crucial role in the gender-specific differences observed in gastrointestinal disorders and their response to hormonal changes.

The higher expression of these receptors in females may reflect an evolutionary adaptation to support the increased metabolic demands and immune modulation required during pregnancy [30]. This gender-specific pattern could have important implications for understanding the higher prevalence of certain gastrointestinal disorders in women and the potential protective effects of progesterone in maintaining gut health [31].

Recent studies have shown that sex hormones can modulate the gut microbiome composition, which in turn affects gut barrier function and immune responses [32]. This interaction between sex hormones and the microbiome could provide additional insights into the gender-specific differences we observed in progesterone receptor expression.

### 3.3. Influences of Lifestyle Factors

Our findings regarding dietary patterns present an intriguing paradox. While previous studies have demonstrated significant dietary influences on gut physiology [33], our study also found a significant impact on progesterone receptor expression, but only in females. This discrepancy may stem from the less extreme dietary modifications in our cohort or the complex interplay between diet, microbiota, and hormonal regulation.

The age-related patterns we observed, particularly the decline in receptor expression after 60, underscore the importance of hormonal changes in gut physiology. This decline may contribute to the increased susceptibility to gastrointestinal disorders observed in older populations. The lack of significant correlation between age and PGRMC1 expression suggests that this receptor may be modulated by different mechanisms, potentially involving oxidative stress or inflammatory processes.

Recent research has highlighted the role of dietary components in modulating gut hormone signaling. For example, polyphenols found in fruits and vegetables have been shown to interact with nuclear receptors, including progesterone receptors, and may influence gut health [34]. These findings suggest that while we did not observe significant effects of diet on receptor expression in our study, more nuanced dietary interventions or specific bioactive compounds may have an impact.

### 3.4. Microbiome Interactions

The correlation between BMI and receptor expression underscores the obesity-microbiota-receptor expression paradigm, at least in females. Our findings suggest that BMI alone may not capture the complex biological processes influencing receptor expression. The microbiome’s role in hormonal modulation becomes particularly evident when considering age-related changes. The decline in bacterial diversity and increase in pro-inflammatory microbes with aging [35] may contribute to the reduced functionality of progesterone receptors we observed in older individuals. This age-related dysbiosis could impair the gut’s ability to respond appropriately to hormonal signals, potentially contributing to the development of gastrointestinal disorders [36].

Recent studies have shown that the gut microbiome can produce neuroactive compounds that influence the ENS and CNS [37,38]. These findings suggest that the microbiome may not only modulate hormone receptor expression but also directly influence neuronal function in the gut [39].

### 3.5. Therapeutic Implications for Parkinson’s Disease

The neuroprotective potential of progesterone in PD is particularly compelling. Our findings suggest that progesterone receptors in the ENS could modulate inflammatory processes and oxidative stress, both of which are key factors in PD pathogenesis [40,41]. The identification of mPRα as a key mediator of neuroprotection opens exciting possibilities for developing targeted therapies [42]. The gender differences in PD prevalence further support the potential of hormone-based therapies, particularly for women experiencing hormonal changes during menopause. The protective effects of progesterone may be particularly relevant for women in this life stage, as decreasing hormone levels correlate with increased susceptibility to neurodegenerative diseases [43].

Recent studies have shown that progesterone can modulate the gut–brain axis, potentially influencing PD progression [27,44]. This interaction between the ENS and CNS highlights the importance of considering the gut in the development of PD therapies. Targeting progesterone receptors in the ENS may offer a novel approach to modulating PD pathology through the gut–brain axis.

### 3.6. Future Directions

While our study provides valuable insights, several questions remain. The clinical relevance of these findings for PD and other neurodegenerative diseases needs to be explored through longitudinal studies. The development of selective progesterone receptor modulators that target specific signaling pathways could offer new therapeutic options. Combination therapies that integrate hormonal modulation with existing PD treatments may prove particularly effective, especially when tailored to individual patient characteristics. These personalized approaches could consider factors such as gender, age, and hormonal status to optimize treatment outcomes.

Recent advances in single-cell sequencing technologies could provide more detailed insights into the cellular distribution of hormone receptors in the ENS [45]. These techniques could help identify specific cell populations that express progesterone receptors and their functional roles in gut physiology.

### 3.7. Study Limitations

We acknowledge several limitations in our study. The use of tissue samples from patients with pre-existing bowel diseases may have influenced our findings, though it provided a clinically relevant context. The cross-sectional design prevented us from establishing causality or tracking longitudinal changes. The absence of direct microbiome analysis limited our ability to explore these complex interactions.

While our study provides valuable insights into the expression of progesterone receptors in the myenteric plexus, the potential role of these receptors in the submucosal plexus warrants further investigation. The submucosal plexus is closer to the mucosa and may be more directly involved in interactions with the microbiome. Future studies should aim to include the submucosal plexus to provide an even more detailed understanding of progesterone receptor distribution and its potential roles in the intestine.

Despite these limitations, our study significantly advances the understanding of progesterone receptor expression in the human ENS and provides a robust foundation for future research. The comprehensive dataset we have generated offers new perspectives on potential therapeutic approaches for neurodegenerative diseases and highlights the importance of considering hormonal factors in gut physiology.

## 4. Materials and Methods

### 4.1. Tissue Preparation and Cryosectioning

All human tissue samples were obtained in accordance with ethical guidelines and regulatory standards. Informed consent was obtained from all participants, and samples were collected during planned surgical procedures. To ensure anonymity, all samples were anonymized prior to analysis. The study was approved by the relevant ethics committee, and all procedures were performed in compliance with the principles of the Declaration of Helsinki. For subsequent analyses, tissue samples were categorized according to patient age, with a cutoff at 60 years, whereby patients older than 60 years were considered postmenopausal.

Human intestinal segments (jejunum, ileum, colon) were either fixed in 4% paraformaldehyde (PFA) for immunofluorescence staining or stored in phosphate-buffered saline (PBS) for RT-PCR and Western blot analysis. For cryosectioning, each intestinal segment was placed in 4% PFA in the operating room and stored at 4 °C for 48 h. After thorough washing with PBS, the tissue was cut transversely into 1–2 cm pieces and incubated in a 30% sucrose solution for another 48 h, followed by freezing in isopentane at −40 °C.

The cryostat (CryoStar NX50, Thermo Scientific, Darmstadt, Germany) was cleaned with NaOH-EDTA (0.1 M NaOH, 1 mM EDTA). The frozen tissue was tempered at −20 °C, with the temperature of the microscope slides maintained between −18 °C and −16 °C for 45 min. Each segment was stabilized with a tissue freezing medium (#2309309815, Tissue-Tek^®^ O.C.T Compound, Sakura Finetek, CA, USA) to prevent distortion during sectioning. Precise adjustment of the sample holder allowed for even sectioning of the tissue layers, which were cut in the transverse plane at a thickness of 14 μm. Sections were checked for proper orientation using light microscopy (BZ-X800, Keyence Corporation, Osaka, Japan) before being placed on special slides and dried at room temperature for 10 min.

### 4.2. Immunofluorescence Staining

After three 5 min washes with PBS, the tissue was permeabilized with 0.3% Triton-X-100 (#T8532; Sigma-Aldrich, St. Louis, MO, USA) in PBS for 15 min, followed by three 5 min PBS washes. Cryosections were outlined with Pap-Pen (#MKP-2, Kisker Biotech GmbH & Co. KG, Steinfurt, Germany) to limit antibody usage. Non-specific binding sites were blocked with donkey serum (S30-M, Sigma-Aldrich, St. Louis, MO, USA; 1:50 in PBS) for 30 min, followed by three 5 min PBS washes. Sections were then incubated overnight with the primary antibody at 4 °C. To visualize the progesterone receptors, either a goat-derived PGRMC1 antibody (Anti-PGRMC1-antibody, ab48012; 1:100 in PBS) or a mouse-derived PR-A/B antibody (PR-AT 4.14, Thermo Fisher, Darmstadt, Germany, # MA1-410; 1:300 in PBS) was used as the specific primary antibody. After another PBS wash (3 × 5 min), secondary antibodies, either donkey anti-goat (Donkey Anti-Goat, ab150129; Abcam, Camebridge, UK; 1:1000 in PBS) or goat anti-mouse (#A11001, Invitrogen, Fisher Scientific GmbH, Schwerte, Germany; 1:500 in PBS), were applied for 2 h, then removed by three 5 min PBS washes. A neuron-specific beta-III-tubulin antibody (#MAB1195, R&D Systems, Singapore, 1:750 in PBS) was used as a second primary antibody to label the neuronal tissue for 2 h at 4 °C, followed by the same washing procedure. The second secondary antibody, donkey anti-mouse (A32744, Invitrogen, Fisher Scientific GmbH, Schwerte, Germany; 1:750 in PBS), was applied for 2 h, followed by sequential washing steps. Cell nuclei were counterstained with DAPI for 30 min (B-1155, Sigma-Aldrich, St. Louis, MO, USA; 1:1000 in PBS). Intensive PBS washes (3 × 5 min) were performed before embedding the samples in a fluorescence mounting medium (F6937, Sigma-Aldrich, St. Louis, MO, USA).

The photographs were taken using a Keyence fluorescence microscope (BZ-X800, Keyence Corporation, Osaka, Japan) with a 20× objective (Plan Apochromat 20×, BZ-PA20, NA 0.75, WD 0.6 mm), and the same exposure time was used for each coverslip in the different channels (DAPI: 1/35 s, PGRMC1 and PR A/B: 1 s, TUJ1: 1 s).

### 4.3. RNA Isolation, cDNA Synthesis, and Quantitative RT-PCR

Human intestinal segments were immediately placed in ice-cold PBS in the operating room. After transport to the laboratory, the mucosa was removed, and the remaining sample was cut into 1–2 cm pieces and frozen in isopentane at −40 °C.

Total RNA was extracted using the NucleoSpin^®^ RNA Kit (REF 740955.50, Macherey-Nagel, Düren, Germany). First, 30 mg of tissue from the myenteric plexus of each human intestinal sample was cut and homogenized. Then the manufacturer’s protocol was followed, with the exception that for the elution of the RNA, 30 μL of nuclease-free water was added directly to the center of the column matrix, followed by centrifugation for 1 min. The isolated RNA (~300 ng/μL) was either immediately processed for further analysis or stored at −80 °C.

For cDNA synthesis, the GoScript™ Reverse Transcription Mix, Oligo(dT) (#A2791, Promega, Walldorf, Germany) was used according to the manufacturer’s protocol. The samples were then stored at 4 °C for immediate RT-qPCR or at −20 °C for later use.

The gene expression of various progesterone receptors in the myenteric plexus was analyzed using RT-qPCR. The expression levels of the housekeeping genes *GAPDH* and *Beta-Actin*, as well as the expression levels of the genes *mPRalpha*, *mPRbeta*, *PGRMC1*, *PRA/B*, and *beta-III-Tubulin* (Table 1), were measured in triplicate and in at least three independent runs. The following components were combined: 10 μL GoTaq qPCR Master Mix (#A6001, Promega, Walldorf, Germany), 1.4 μL of forward primer, 1.4 μL of reverse primer, diluted cDNA (1:10), and ddH_2_O to a final volume of 20 μL. The samples were analyzed using the CFX96 Real-Time PCR Detection System (BioRad, Hercules, CA, USA), which involved an initial heating step at 95 °C for 2 min, followed by 40 amplification cycles of 15 s at 95 °C and 60 s at 60 °C. Melt curves were recorded after each cycle to detect individual PCR products.

### 4.4. Statistical Analysis

For statistical analysis, Microsoft Excel (Microsoft Corporation, Redmond, WA, USA) and GraphPad Prism version 7.0a (GraphPad, San Diego, CA, USA) were used. The 2^−Δct^ method was used to calculate relative gene expression levels, which were then plotted as mean ± standard deviation (SD). Prior to statistical analysis, data were tested for normal distribution using the Shapiro–Wilk test, and statistical outliers were removed using the ROUT method with a Q of 5%. Only groups with a sample size of *n* ≥ 3 were included in the analysis. All in all, 63 human samples were collected, whereof 38 were from male patients and 25 from female patients. We were able to collect 6 samples from Jejunum, 19 from Ileum, 1 from ascending colon, 5 from transversal colon, 15 from descending colon, 14 from sigmoid colon and 3 from rectum. The samples were used for immunohistochemical or molecular biological analysis, or both. Significant differences between groups were assessed using one-way ANOVA followed by Tukey’s post hoc test. Statistical significance is indicated as * *p* < 0.05, ** *p* < 0.01, *** *p* < 0.001, and **** *p* < 0.0001.

## Figures and Tables

**Table 1:** RT-qPCR primers used in this study.

Gene	Forward	Reverse
*mPRα*	5′-CGC TCT TCT GGA AGC CGT ACA TCT ATG-3′	5′-CAG ACG GTG GGT CCA GAC ATT CAC-3′
*mPRβ*	5′-GTC CAT CTG TAC GCT CTC CC-3′	5′-GCA GGC CAT GTG GAC AGA TA-3′
*PGRMC1*	5′-GAC CAA AGG CCG CAA ATT CT-3′	5′-CAG GCA AAA TGT GGC AAG GC-3′
*PR-A/B*	5′-AAA TCT ACA ACC CGA GGC GG-3′	5′-ACT TCT GCT GGC TCC GTA CT-3′
*GAPDH*	5′-GGG GAG CCA AAA GGG TCA TC-3′	5′-ATG ATC TTG AGG CTG TTG TCA TAC T-3′
*Actin* *β*	5′-AAA CTG GAA CGG TGA AGG TG-3′	5′-CTC GGC CAC ATT GTG AAC TTT-3′
*beta III Tubulin*	5′-TCA GCG TCT ACT ACA ACG AGG C-3′	5′-GCC TGA AGA GAT GTC CAA AGG C-3′

## Data Availability

The original contributions presented in this study are included in the article. Further inquiries can be directed to the corresponding author.
